# Physiological and Biochemical Responses of Bicarbonate Supplementation on Biomass and Lipid Content of Green Algae *Scenedesmus* sp. BHU1 Isolated From Wastewater for Renewable Biofuel Feedstock

**DOI:** 10.3389/fmicb.2022.839800

**Published:** 2022-03-29

**Authors:** Rahul Prasad Singh, Priya Yadav, Ajay Kumar, Abeer Hashem, Al-Bandari Fahad Al-Arjani, Elsayed Fathi Abd_Allah, Angélica Rodríguez Dorantes, Rajan Kumar Gupta

**Affiliations:** ^1^Laboratory of Algal Research, Centre of Advanced Study in Botany, Institute of Science, Banaras Hindu University, Varanasi, India; ^2^Botany and Microbiology Department, College of Science, King Saud University, Riyadh, Saudi Arabia; ^3^Plant Production Department, College of Food and Agricultural Sciences, King Saud University, Riyadh, Saudi Arabia; ^4^Escuela Nacional de Ciencias Biológicas, Instituto Politécnico Nacional, Mexico City, México

**Keywords:** *Scenedesmus* sp., sodium bicarbonate, chlorophyll fluorescence, molecular analysis, scanning electron microscopy, biofuel

## Abstract

In the present study, different microalgae were isolated from wastewater environment and evaluated for higher growth and lipid accumulation. The growth adaptability of all the isolated microalgae were tested for carbon source with supplementation of sodium bicarbonate in BG-11 N^+^ medium. Further based on the uptake rate of sodium bicarbonate and growth behavior, microalgal strains were selected for biofuel feedstock. During the study, growth parameters of all the isolates were screened after supplementation with various carbon sources, in which strain *Scenedesmus* sp. BHU1 was found highly effective among all. The efficacy of *Scenedesmus* sp. BHU1 strain under different sodium bicarbonate (4–20 mM) concentration, in which higher growth 1.4 times greater than control was observed at the concentration 12 mM sodium bicarbonate. In addition, total chlorophyll content (Chl-a + Chl-b), chlorophyll fluorescence (Fv/Fm, Y(II), ETR max, and NPQmax), and biomass productivity were found to be 11.514 μg/ml, 0.673, 0.675, and 31.167 μmol electrons m^−2^ s^−1^, 1.399, 59.167 mg/L/day, respectively, at the 12 mM sodium bicarbonate. However, under optimum sodium bicarbonate supplementation, 56.920% carbohydrate and 34.693% lipid content were accumulated, which showed potential of sodium bicarbonate supplementation in renewable biofuel feedstock by using *Scenedesmus* sp. BHU1 strain.

## Highlights

- The physiological and biochemical composition of microalgae affected by sodium bicarbonate supplementation.
- Photosynthetic efficiency and microalgal biomass of isolate was found to be maximum at the 12 mM bicarbonate concentration.
- At 12 mM sodium bicarbonate, the maximum total chlorophyll content and biomass productivity were reported to be 11.514 g/ml and 59.167 mg/L/day, respectively.
- 56.92% carbohydrate and 34.69% lipid accumulated under optimum sodium bicarbonate supplementation.
- Strain *Scenedesmus* sp. BHU1 can be used as a potent renewable biofuel feedstock.

## Introduction

In recent years, search of an alternative renewable energy resource is of great concern worldwide. The rapid industrialization and the needs of a burgeoning human population put extra pressure on energy resources. However, the limited stock of fossil fuels, regular price hiking of conventional energy resources, and release of toxic effluents after utilization need an immediate search of an alternative that must be economic, ecofriendly, and renewable in nature. In this regard, biofuel appears as a suitable alternative in terms of sustainability, reducing greenhouse gas emissions, and controlling changing climatic conditions. Generally, biofuels are classified into first, second, third, and fourth generations and objectives of all these are to meet the needs of global energy requirements in sustainable ways (Khoo et al., 2020; Mat Aron et al., 2020).

In the recent past, different microalgal strains have been frequently utilized as renewable energy feedstock with potential to replace traditional fuels (Behera et al., 2015; Cheah et al., 2018). Microalgae are photosynthetic organisms that use CO_2_ and sunlight to develop and produce various value-added products such as carotenoids, fatty acids, and natural antioxidants, which are directly or indirectly utilized for human welfare (Borowitzka, 1992). However, for biofuel production, various higher plants have been reported to date, but high photosynthetic rate, greater nutrient uptake ability, short generation time, easy growth conditions, higher biomass, and lipid content make microalgae preferred over the higher plants (Karemore et al., 2013; Rawat et al., 2013; Arenas et al., 2017). The biomass, carbohydrate, and lipid productivity are crucial factors to optimize or enhance the economic viability of microalgae-based biofuels. Changes in medium ingredients or growing conditions can easily increase the biomass content of microalgae resulting in a greater yield of value-added products (Vijay et al., 2021; Siddiki et al., 2022).

Biofuel production from microalgal biomass is still in the initial stages and the industrial growth is largely dependent on the viability of large-scale microalgal cultivation. To ensure the economic feasibility of microalgal cultivation and biofuel production, selection of suitable strain, cultivation methods, nutrient supply, and feasible source of water (Borowitzka and Vonshak, 2017). Even though microalgae have high oil content in their biomass, their ability to synthesize substantial amounts of lipid is often strain-specific and varies with surrounding environmental conditions. In a study, Parichehreh et al. (2021) evaluated several factors including light intensity, temperature, salinity, NaHCO_3_, CO_2_, and other nitrogen sources on the growth, biomass, and lipid production of *Chlorella* sp. Authors reported light intensity and temperature have significant effects on algal growth. However, bicarbonate as carbon and ammonium as nitrogen source play significant roles in biomass and lipid production compared to CO_2_ and nitrate, respectively.

Wastewater microalgal strains are referred to as a promising option for biofuel generation, due to their tolerance to climatic and environmental changes (Priyadharshini et al., 2021; Renuka et al., 2021). Several strains of freshwater microalgae *Scenedesmus* and *Chlorella* have been documented as potential candidates for the large-scale production of biofuel owing to its high biomass productivity and favorable biochemical composition. In addition, the ability to modulate their metabolic productivity under varying nutritional conditions, which showed significant morphological variability and phenotypic plasticity in response to changing environmental factors (Chung et al., 2018; Yun et al., 2019; Vijay et al., 2021). Microalgal biomass production on a large-scale is difficult as is microalgal strains that can develop robustly under stress conditions. Although under ideal stress conditions, various microalgal species have been examined and reported to produce 30%–50% lipid of dry cell weight (Maheshwari et al., 2020; Han et al., 2021). About 60 microalgal species have been thoroughly studied to produce enormous amounts of biomass and high biofuel yield (Tripathi et al., 2015).

The *Scenedesmus* sp. is a microscopic unicellular microalga that can be found in both fresh and effluent streams. The microalga *Scenedesmus* sp. is a significant biofuel feedstock because of its capacity to grow in a variety of carbon-rich habitat with high biomass yield and lipid content (Mata et al., 2012). Previously, several species of *Tetradesmus/Acutodesmus* which belong to the Scenedesmaceae family have been reported as promising candidates for biofuel production due to their high lipid content and suitable fatty acid profile (Ferrigo et al., 2015; Ismagulova et al., 2018; Umetani et al., 2021). However, limited information is available for *Scenedesmus* sp. Therefore, we are selecting *Scenedesmus* sp. to evaluate its physiological and biochemical responses to bicarbonate supplementation and to use it for various biotechnological applications.

Therefore, the current research has been designed to isolate different microalgal strains from wastewater environment. Furthermore, screening of different microalgal strains have been carried out under supplementation of different ranges of sodium bicarbonate as a carbon source and determine the physiological and biochemical responses in terms of biomass and lipid content to bicarbonate supplementation from microalgae as a biofuel feedstock.

## Materials and Methods

### Isolation and Cultivation of Microalgae

The samples of microalgae were collected aseptically from the wastewater environment of Banaras Hindu University (BHU) campus, Varanasi, India. A total of 10 ml of microalgal culture was inoculated in 250 ml Erlenmeyer conical flasks with 100 ml BG-11 N^+^ broth medium at pH 7.4 (Rippka et al., 1979). The cultured samples were disseminated on BG-11 N^+^ agar plate and incubated in an artificial photoperiod of 14 h light and 10 h dark, illuminated by white fluorescent tube light (55 μ mol m^−2^ s^−1^) at 28 ± 2°C and each flask was shaken at 50 rpm continuously. The separate colonies that appeared on agar plate were picked and transferred to a similar medium for purification using BG-11 N^+^ broth and agar plates in a sequential culture. The procedure was repeated until axenic microalgal cultures were achieved.

### Screening of Potential Microalgal Isolates

The microalgal isolates capable of using high inorganic carbon as sodium bicarbonate from the aquatic environment were screened. The selection of isolates was performed in BG-11 N^+^ medium with 12 mM sodium bicarbonate and the growth was analyzed in terms of optical density (OD) every alternate day until the 16 days at 680 nm wavelength by UV-2600 UV–VIS Spectrophotometer, SHIMADZU (Japan). The microalgal isolates that respond better at 12 mM sodium bicarbonate were transferred to a 250-ml flask with a 100-ml working volume for further study.

### Morphological Identification of Microalga by Scanning Electron Microscopy

On the center of a coverslip (22 mm × 22 mm Blue Star) a drop of exponentially growing microalgal suspension (0.7 OD) was placed and dried in air. It was then chemically fixed with 3% (w/v) glutaraldehyde and left overnight before being rinsed thrice in distilled water and dehydrated with a graded series of ethanol (30%, 50%, 80%, 90%, 95%, and 100%) and air dried. In a quorum Sc 7620 sputter coater air dried sample was coated with a 30 Å thick gold palladium coating for 9 min. Scanning electron microscopy, EVO18 Research ZEISS (Germany) was used to examine coated materials at 15 and 5 kV (Sadiq et al., 2011).

### Molecular Identification of the Potential Microalgal Isolate

Based on a microscopic study the microalgal strain was initially recognized morphologically. After that 18S rDNA sequencing was used to confirm the identity of the microalgal isolate. The manual DNA isolation technique was used to isolate genomic DNA from a microalgal strain (Aboul-Maaty and Oraby, 2019). The 18S rDNA was amplified using PCR with forward 5′-GCTTAATTTGACTCAACACGGGA-3′ and reverse 3′-AGCTATCAATCTGTCAATCCTGTC-5′ primers (Gargas and DePriest, 1996). A reaction mixture containing 0.2 mM deoxynucleoside triphosphates, 1.5 mM MgCl_2_, 0.5 mM of each primer and 1.25 units of *Taq* DNA polymerase were used for amplification. The following polymerase chain reaction procedure was used: 94°C for 3 min, 35 cycles of 1 min at 94°C, 1 min at 59°C, and 1 min at 72°C (Valiente Moro et al., 2009). The 400–900 bp amplicon was gel eluted and the amplicon was sequenced using the Sanger technique of DNA sequencing (Sanger et al., 1977). The outcomes of the sequencing were combined and compared with existing National Center for Biotechnology Information (NCBI) data base of gene bank.

### Phylogenetic Analysis

The 18S rDNA sequence of isolated microalga was matched with previously reported sequences of other intimately related microalgal species to determine its phylogeny. Using the nucleotide BLAST tool, the 18S rDNA gene sequence obtained in our study and NCBI reference sequences were downloaded and aligned using Clustal-W multiple sequence alignment. The Neighbor-Joining approach was used to create a phylogenetic tree and the Maximum Composite Likelihood technique was used to calculate the evolutionary distances (Saitou and Nei, 1987; Tamura et al., 2004). Bootstrap analysis based on 1,000 replications was used to determine the tree resilience. The MEGA 11 software was used to conduct evolutionary analyses (Tamura et al., 2021).

### Growth Characterization

The growth was measured at 680 nm OD and the pigment content (chl-a, chl-b, and carotenoid), chlorophyll fluorescence, and biomass of each culture growing in different concentrations of sodium bicarbonate ranges from 4 to 20 mM were measured in terms of their growth.

### Determination of Photosynthetic Pigments

To assess pigment content, 2 ml microalgal culture was centrifuged for 5 min at 10,000 rpm, the pellet was dissolved in 99.9% methanol and kept at 4°C overnight in the dark. The absorbance of the supernatant was taken at 470, 652.4, and 665.2 nm using a UV-2600 UV–VIS Spectrophotometer, SHIMADZU (Japan) and the pigment content was calculated using the following formulas (Lichtenthaler, 1987).


$$\mathrm{Chlorophyll}\;a;\mathrm{Chl-a}\;\left(\mu g/\mathrm{ml}\right)=16.72\;A_{665.2}-9.16\;A_{652.4}$$

$$\mathrm{Chlorophyll}\;b;\mathrm{Chl-b}\;\left(\mu g/\mathrm{ml}\right)=34.09\;A_{652.4}-15.28\;A_{665.2}$$

$$\mathrm{Carotenoid}\;\left(\mu g/\mathrm{ml}\right)=\left(1000\;A_{470}-1.63\;\mathrm{Chl-a}-104.9\;\mathrm{Chl-b}\right)/221$$

### Chlorophyll Fluorescence Analysis

Chlorophyll fluorescence (ChlF) used as a non-destructive marker of photosynthetic performance and various photosynthetic parameters like the maximum photochemical quantum efficiency (Fv/Fm), effective photochemical quantum yield [Y(II)], the maximum electron transport rate (ETRmax), and maximum non-photochemical quenching (NPQmax) of photosystem II (PSII). These parameters were measured using a KS-2500 pulse-amplitude modulation (PAM) device (Heinz Walz GmbH D-91090 Effeltrich, Germany) and computed using PAM software (PamWin-3). The 0.5 ml aliquot of dark-adapted (20 min) culture was poured into a glass cuvette attached to a PAM fluorometer with a micromagnetic stirrer. Maximum chlorophyll fluorescence (Fm) was measured using high intensity saturating pulses of more than 3,000 mol m^−2^ s^−1^, while minimal chlorophyll fluorescence (Fo) was measured using a light-emitting diode providing 0.001 mol m^−2^ s^−1^. A range of photosynthetic active radiation (PAR) (0–2,973 mol m^−2^ s^−1^) was used for light curve analysis of samples. The maximum electron transport rate (ETR) was determined to be 984 mol m^−2^ s^−1^.

### Biomass Productivity

At the 14th day, dry cell weight of each culture treated with different concentrations of sodium bicarbonate (4–20 mM) was measured to estimate biomass. To achieve constant weight, 2 ml of each microalgal culture was centrifuged and transferred to pre-weighted Eppendorf tube for drying in the oven at 70°C. The biomass content (c) was calculated using formula *c* = *m*/0.02 L (Chen et al., 2021), where m is the weight of the dry cell pellet.

### Determination of Protein, Carbohydrate, and Lipid Content

The Lowry method was used to estimate the protein content of the microalgal biomass (Lowry et al., 1951) and the anthrone method was used to determine total carbohydrate content (Loewus, 1952). The anthrone reagent was prepared by dissolving 0.2 g anthrone in 100 ml chilled 95% H_2_SO_4_. There was 1 ml stationary phase microalgal culture pelleted and treated with 1 ml 1 N NaOH. The total carbohydrate was determined using 100 μl of supernatant, 900 μl of deionized water, and 4 ml anthrone reagent and incubated at room temperature for 10 min in the dark, a greenish-blue color appeared, and absorbance was measured at 625 nm. For the standard, glucose stock was used.

The Bligh and Dyer method was used to extract total lipids content from microalgae (Bligh and Dyer, 1959). For lipid extraction, stationary phage grown microalgal cells were collected, after centrifugation for 6 min at 8,000 rpm. The extraction solvent chloroform:methanol:water (2:1:1 v/v/v) was added in the microalgal cells and disrupted with a Sonics vibra cell sonicator for 10 min. The mixture sample was centrifuged and the chloroform layer containing lipid fraction was isolated and evaporated using a speed vacuum centrifuge evaporator and the total lipid content was determined gravimetrically using formula *c* (%) = (*m_c_* − *m_e_*)/*m_a_* × 100%, where *m_c_* is the weight of the tube containing the dry total lipids, *m_e_* is the weight of the empty collection tube, and *m_a_* is the weight of the dry cell pellet used for extraction.

### Qualitative Analysis of Lipid by BODIPY 505/515 and Nile Red Dyes

Two lipophylic dyes BODIPY 505/515 and Nile Red (NR-47353) powder (0.250 μg) were dissolved in 1 ml of acetone, which was used as the stock solution to detect the presence of neutral lipid in microalgal cells (Singh et al., 2019). Stationary phase microalgal cells (2 ml) were collected by centrifugation and washed three times with 1x Phosphate Buffer Saline (PBS). There was 5 μl microalgal suspensions taken on the center of slide and 2 μl stock solution was added and incubated for 5 min in the dark. The stained microalgal cells were observed by fluorescent microscopy (Nikon ECLIPSE 90i, United States) using 20x objective lens with a common exposure period for all images, having NIS-Elements AR 4.0 microscope imaging software (Nikon Instech Co. Ltd., Japan) at 465–495 and 590–650 nm for green and red channel excitation emission wavelengths, respectively.

### Statistical Analysis

The consequences were presented as three replicate averages with standard errors. Using SPSS software version 21, the significance of differences between treatment was examined using the Tukey’s-b *post hoc* test at a *p* < 0.05 probability threshold.

## Results and Discussion

### Isolation and Screening of Microalgal Strains

**Figure 1:**
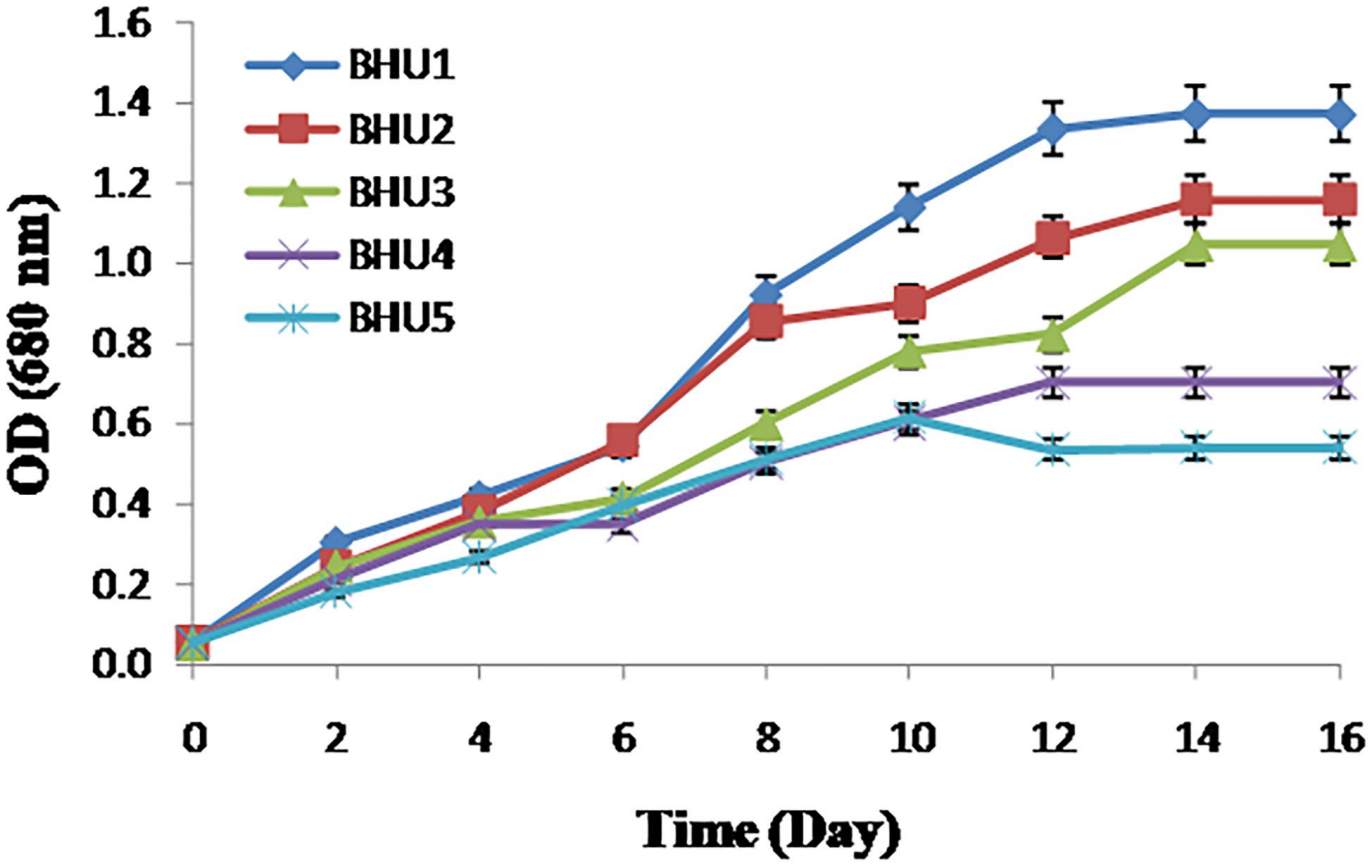
Growth measurement in terms of optical density of different algal strains grown in BG − 11 N^+^ medium supplemented with 12 mM NaHCO_3_. The data represents mean ± SE (*n* = 3).

Using standard microbiological procedures, five axenic microalgal isolated strains namely BHU1, BHU2, BHU3, BHU4, and BHU5 capable of thriving in inorganic enriched environments were identified from wastewater habitat. Among the five isolates, the strain BHU1 grew vigorously after supplementation of 12 mM sodium bicarbonate than BHU2, BHU3, BHU4, and BHU5 (Figure 1), which indicates that it can effectively absorb excess inorganic carbon given as bicarbonate and sequester it into increased biomass. *Scenedesmus* sp. along with other microalgae is commonly found in wastewater environment (Apandi et al., 2017; López-Pacheco et al., 2021). At such ecological niche, carbonate and bicarbonate ions are the primary sources of inorganic carbon, hence microalgal group inhabitant of wastewater, naturally adopted to survive under high inorganic carbon concentrations and possibly improve inorganic carbon uptake as well as enhancement in biomass and lipid productivity (Lee et al., 2014; Pandey et al., 2019).

### Identification of Isolated Microalga Using Microscopic and Molecular Techniques

**Figure 2:**
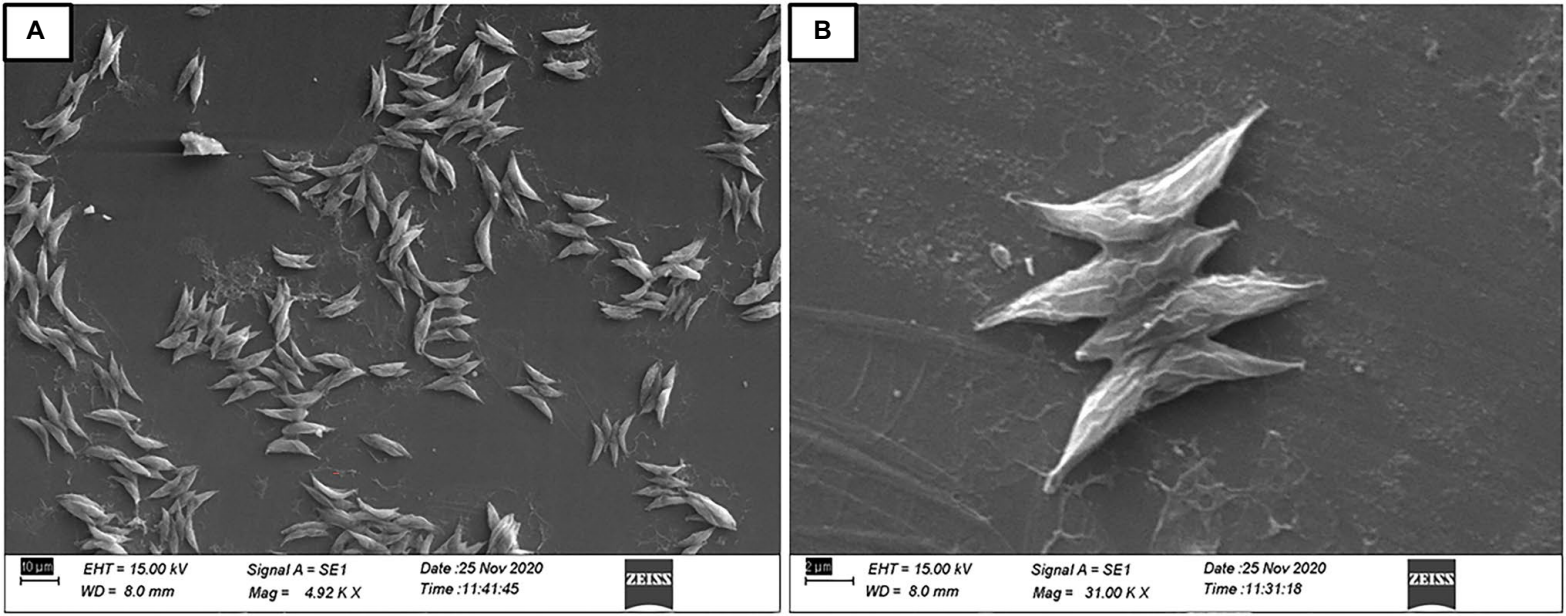
Scanning electron micrograph of isolated strain *Scenedesmus* sp. BHU1 from aquatic habitat of Banaras Hindu University. **(A)** 4.92 KX and **(B)** 31.00 KX magnifications were used to capture the image by scanning electron microscopy (EVO18 Research ZEISS).

**Figure 3:**
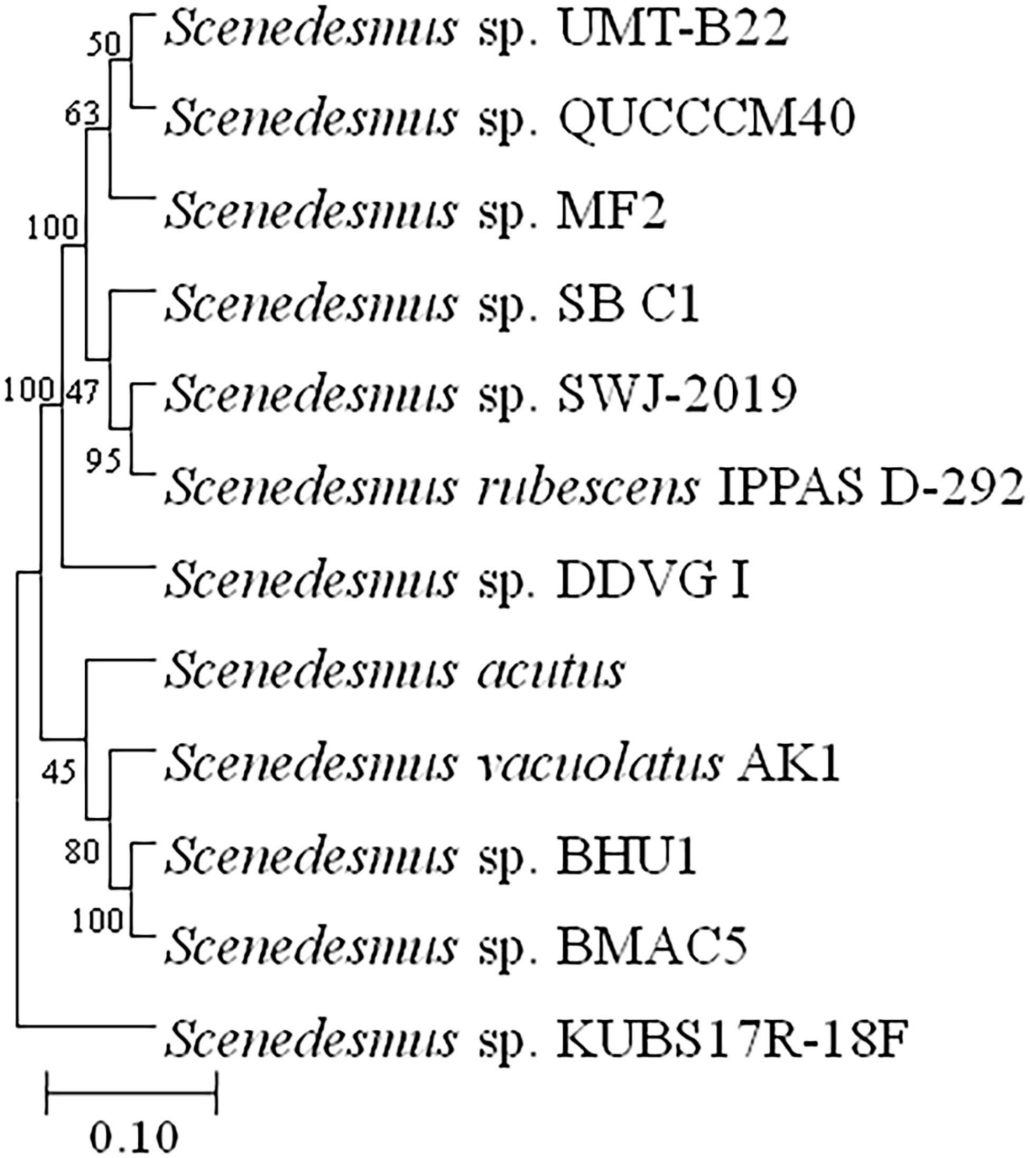
Phylogenetic tree and evolutionary relationships of microalga *Scenedesmus* sp. BHU1 based on 18S rDNA gene sequence. The boot strap consensus tree was created using the MEGA 11 software and multiple sequence alignment with the neighbor-joining method.

The purity and shape of prospective microalgal strains were revealed by scanning electron microscopy. The morphological features of the isolate have confirmed its close relationship with the genus *Scenedesmus*. The microalgal strain BHU1 showed green, cylindrical cells with pointed ends that are joined laterally into 2–4 cell colonies (Figure 2), pair-wise alignments and pointed end gave the closest match with *Scenedesmus* sp. (Guiry and Guiry, 2021), which was later confirmed by 18S rDNA sequence analysis (Gene Bank Accession Number MZ788699) by confirming 99.77% phylogenetic identity with *Scenedesmus* sp. as described in Figure 3. *Scenedesmus* belongs to Scenedesmaceae family, which are already reported as a probable source for biofuel production in recent years with limited information (Pancha et al., 2015; Tripathi et al., 2015; Kumar et al., 2021).

### Effect of Sodium Bicarbonate Supplementation on Growth of Microalgae

**Figure 4:**
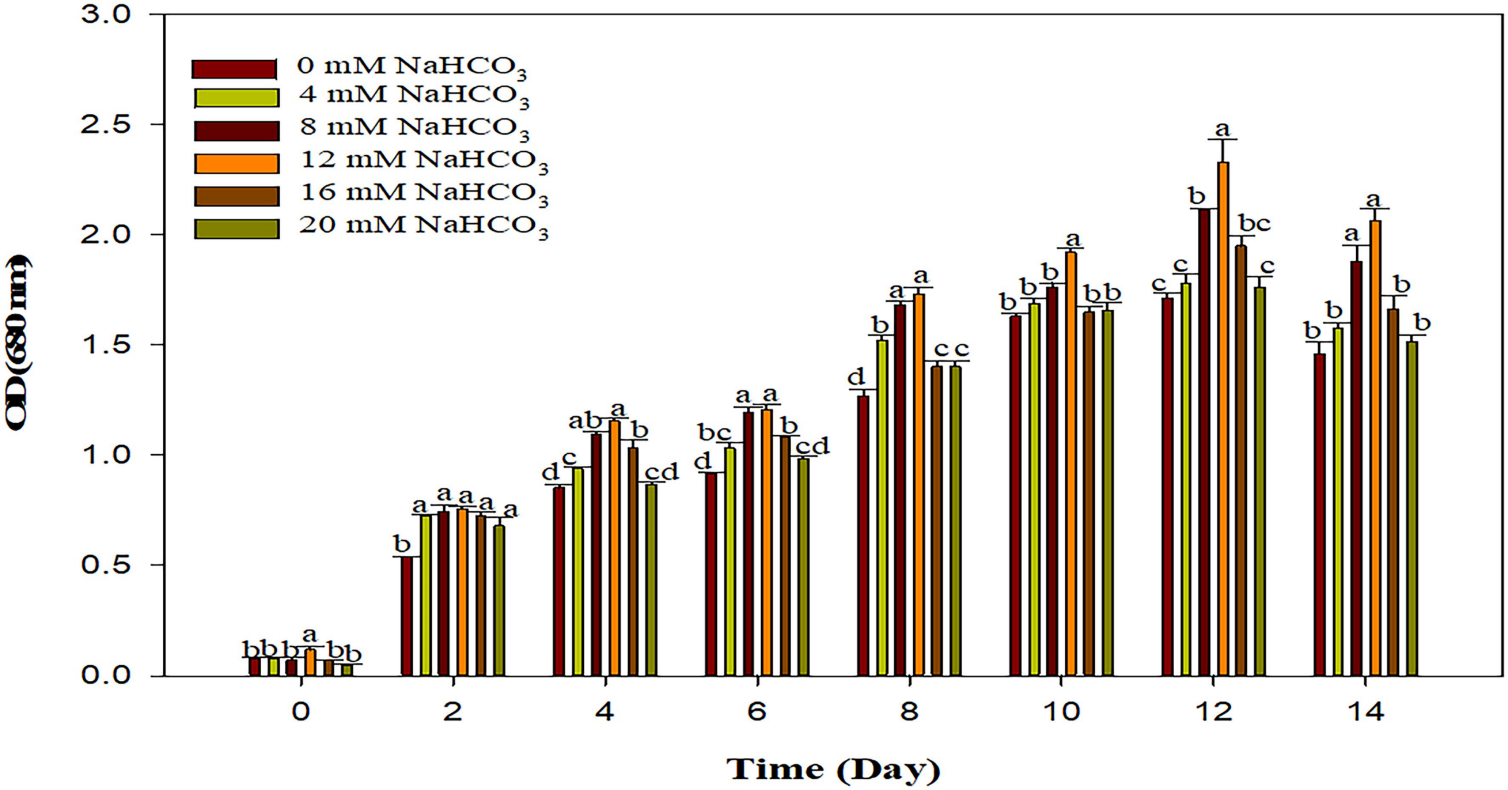
Effect of different sodium bicarbonate concentrations with time (day) on growth pattern of microalga *Scenedesmus* sp. BHU1. Bars are mean ± SE of three replicates. Bars adhered with different alphabets (a, b, c, and d) are statistically significant at *p* < 0.05 (Tukey’s-b *post hoc* test).

The supplementation of sodium bicarbonate in the range of 4–12 mM, significantly enhances the growth of *Scenedesmus* sp. BHU1 as compared to control, while a higher concentration (16–20 mM) of sodium bicarbonate significantly reduced growth due to enhanced concentration of sodium ions in the growth culture with increasing sodium bicarbonate supplementation has been shown (Figure 4). The highest significant growth was observed in the case of 12 mM sodium bicarbonate (2.331 ± 0.179), which was almost 1.3 times higher than the control grown culture (1.714 ± 0.037). The previous research with *Chlorella vulgaris* reported that adding sodium bicarbonate (1 g/L) in growing culture media increased the optical density to 0.5 (Borowitzka, 2005).

The growth of algae was inhibited after higher supplementation of sodium bicarbonate (16–20 mM) and this may be because introducing sodium bicarbonate in such quantities causes a rapid increase in pH, resulting in a slightly alkaline solution, which is unfavorable for microalgal growth. Previous studies have found that at the 75 ppm bicarbonate the *Chlorella* sp. grows at its fastest and has the highest lipid content suggesting it could be used as a biodiesel feedstock (Devgoswami et al., 2011). The result in terms of optical density revealed that elevated concentration of carbon as bicarbonate to the microalgal photosynthetic activity can boost the rate of microalgal reproduction and ultimately growth. The maximum specific growth rate was greater when 1 g L^−1^ bicarbonate was supplemented to green microalgae *C. vulgaris* and *Dunaliella salina* grown culture (Mokashi et al., 2016; Srinivasan et al., 2018). The photosynthetic rate, biomass yield, and fatty acid production of a green microalga *Tetradesmus wisconsinensis* were also improved by an inorganic carbon regime (Umetani et al., 2021). Our research findings also suggest the supplementation of optimum concentration of bicarbonate can boost microalgal growth and biofuel production.

### Effect of Sodium Bicarbonate Supplementation on Photosynthetic Pigment

Based on pigment content, the microalga *Scenedesmus* sp. BHU1 was examined for its growth response after different concentrations of sodium bicarbonate supplementation. The addition of sodium bicarbonate in the growth culture significantly enhanced the photosynthetic pigments including Chl-a, Chl-b, and carotenoid in the microalga *Scenedesmus* sp. BHU1 (Table 1). The Chl-a, Chl-b, and carotenoid content was significantly (*p* < 0.05) increased, when the sodium bicarbonate concentration was increased from 4 to 12 mM, except with 20 mM sodium bicarbonate supplementation, where the Chl-a and Chl-b content was low and carotenoid was high. The highest amount of Chl-a (8.321 ± 0.828 μg/ml) was found in 12 mM sodium bicarbonate supplied culture, which was almost 1.5 times higher than the control grown culture 5.416 ± 0.127 μg/ml (Pancha et al., 2015). Similar to our result, *Scenedesmus* sp. CCNM 1077 had a higher level of Chl-a (6.11 ± 1.14 μg/ml) in a 0.9 g/L sodium bicarbonate enriched culture, which was almost 1.1 times higher than the control grown culture. 5.18 ± 0.32 μg/ml (Pancha et al., 2015). In another study, *Nannochloropsis salina* and *Tetraselmis suecica* were cultivated with 1 g/L bicarbonate supplementation, their Chl-a concentration increased 1.9 and 2.2 times as compared to control grown culture (White et al., 2013). The enhancement in carotenoid was dose dependent with addition of sodium bicarbonate. The highest amount of carotenoid (3.236 ± 0.057 μg/ml) was found in 20 mM sodium bicarbonate supplemented grown culture followed by 16 mM (2.986 ± 0.091 μg/ml) and 12 mM (2.981 ± 0.072 μg/ml) sodium bicarbonate supplemented cultures. Further, sodium bicarbonate addition (up to 12 mM) to the growing culture increased the total chlorophyll (a + b) content (11.514 ± 0.778 μg/ml). In our study, addition of 20 mM sodium bicarbonate to the growing culture lowered the ratio of Chl a/b (0.3-fold) and carotenoid/total Chl (0.9-fold) as compared to the control for microalga *Scenedesmus* sp. BHU1. The decreased pigment ratio can be explained by the fact that adding sodium bicarbonate to such amounts causes a rapid increase in pH, resulting in an alkaline environment, which is unfavorable for microalgal growth. In similar research, there was no discernible difference in pigment ratio when *T. suecica* was cultured with sodium bicarbonate supplementation, whereas *N. salina*, carotenoid/Chl-a ratio increased when sodium bicarbonate in the growth culture was increased (White et al., 2013). Because of this, alga could have CO_2_ concentrating mechanism, and contribute a role in the pH balance. Previous research has found that at 75 ppm bicarbonate, the *Chlorella* strain grows at its fastest and has the highest lipid content, suggesting that it could be used as a biodiesel feedstock (Devgoswami et al., 2011). In our study, *Scenedesmus* sp. BHU1 grew optimally at 12 mM sodium bicarbonate supplementation, and it belongs to the same group of previously investigated green microalgae.

**Table 1:** Effect of different concentration of sodium bicarbonate on photosynthetic pigment content of *Scenedesmus* sp. BHU1.

NaHCO_3_ (mM)	Chl-a^*^ (μg/ml)	Chl-b^**^ (μg/ml)	Caro^***^ (μg/ml)	Chl a + b (μg/ml)	Chl a/b	Caro/Chl a + b
0	5.416 ± 0.127^c^	1.307 ± 0.134^b^	2.478 ± 0.024^c^	6.723 ± 0.198^e^	4.265 ± 0.588^a,b^	0.369 ± 0.003^a^
4	7.047 ± 0.105^a,b^	1.424 ± 0.115^b^	2.520 ± 0.036^c^	8.471 ± 0.010^d^	5.033 ± 0.523^a^	0.297 ± 0.004^b^
8	7.533 ± 0.183^a^	2.729 ± 0.248^a,b^	2.940 ± 0.058^b^	10.263 ± 0.068^b^	2.821 ± 0.336^a,b^	0.286 ± 0.007^b,c^
12	8.321 ± 0.828^a^	3.192 ± 0.904^a^	2.981 ± 0.072^a,b^	11.514 ± 0.778^a^	3.500 ± 1.609^a,b^	0.259 ± 0.004^c^
16	7.149 ± 0.132^a,b^	4.539 ± 0.123^a^	2.986 ± 0.091^a,b^	11.687 ± 0.043^a^	1.579 ± 0.072^a,b^	0.255 ± 0.007^c^
20	5.648 ± 0.054^b,c^	3.961 ± 0.311^a^	3.236 ± 0.057^a^	9.609 ± 0.286^c^	1.444 ± 0.116^b^	0.337 ± 0.015^a^

Values are mean ± SE, *n* = 3. Values adhered with different alphabets (a, b, c, d, and e) are statistically significant at *p* < 0.05.

* Chl-a: chlorophyll-a.

** Chl-b: chlorophyll-b.

*** Caro: carotenoids.

### Effect of Sodium Bicarbonate Supplementation on Chlorophyll Fluorescence and Photosynthetic Performance

**Figure 5:**
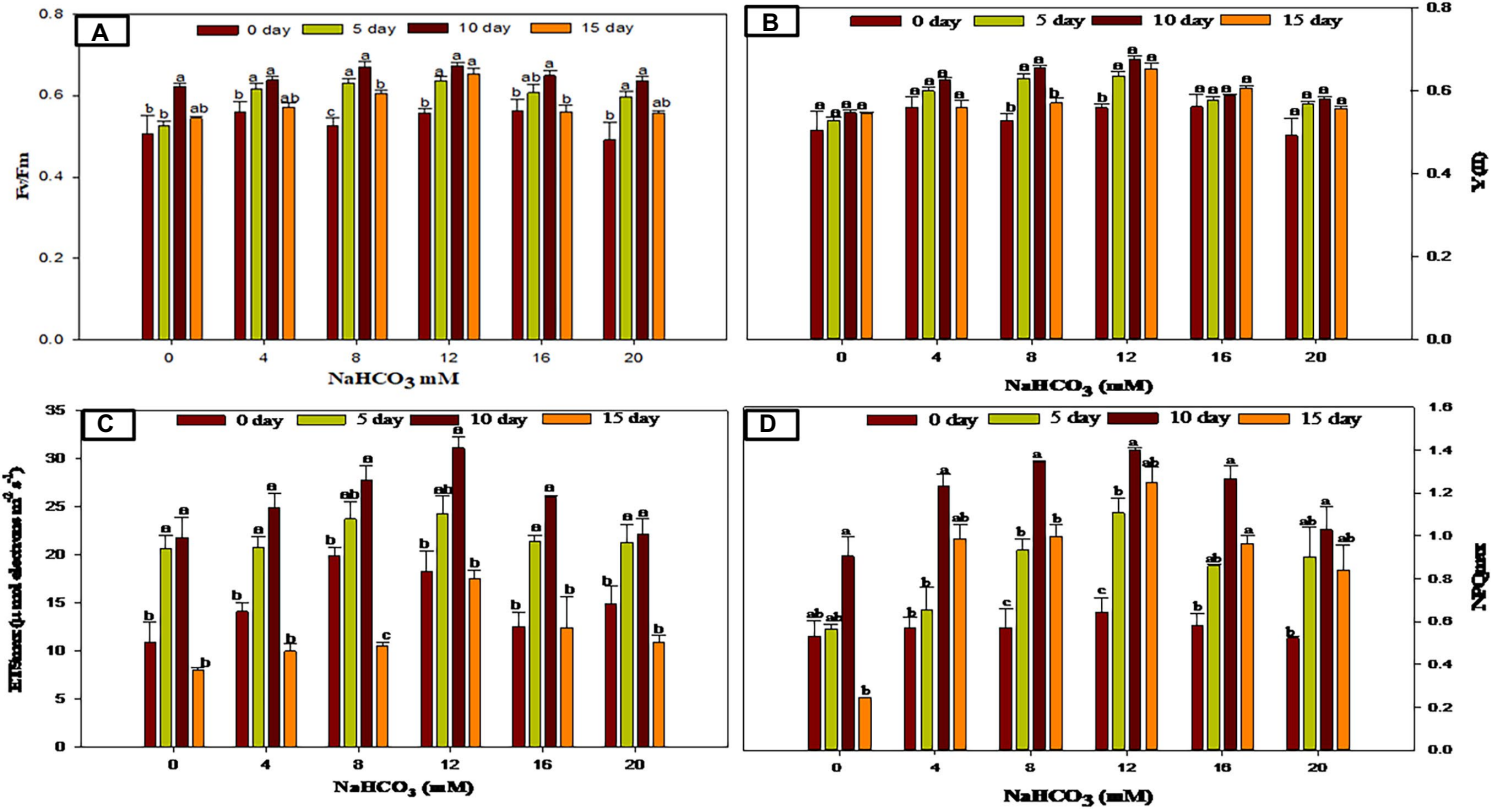
Chlorophyll fluorescence and photosynthetic performance in *Scenedesmus* sp. BHU1 with various NaHCO_3_ concentrations at different days. Bars are mean ± SE of three replicates. Bars adhered with different alphabets (a, b, and c) are statistically significant at *p* < 0.05 (Tukey’s-b *post hoc* test).

Pulse amplitude modulated (PAM) fluorometry is one of the frequently used, non-invasive, and quick procedures that measure the chlorophyll fluorescence variability and photosynthetic activity in microalgae (Baker, 2008; White et al., 2011). The physiological condition of the *Scenedesmus* sp. BHU1 with varied concentrations of sodium bicarbonate was recorded significantly over the full course of the studies using the PAM Flourometer with various parameters such as Fv/Fm, Y(II), ETRmax, and NPQmax (Figures 5A-D). Microalgal cells treated with varied concentrations of sodium bicarbonate ranging from 4 to 12 mM showed a gradually significant (*p* < 0.05) raise in Fv/Fm and Y(II) as compared to the control, showing a good photosynthetic performance in these sodium bicarbonate concentration ranges. The highest Fv/Fm and Y(II) were found in culture grown with 12 mM sodium bicarbonate at 10 days (0.673 ± 0.006, 0.675 ± 0.008) which was 1.2- and 1.2-fold higher than the 12 mM sodium bicarbonate at 0 day of growth culture (0.558 ± 0.010, 0.558 ± 0.010), respectively, and no discernible change (*p* < 0.05) in Fv/Fm and Y(II) was observed beyond 12 mM sodium bicarbonate. In a similar study, the presence of sodium bicarbonate enhanced Fv/Fm and Y(II) in the green alga *Chlorella sorokiniana* (Salbitani et al., 2020). The Fv/Fm and Y(II) rising could suggest increased photosynthetic carbon fixation and metabolic activity. The ETRmax followed the same patterns as Fv/Fm and Y(II). Bicarbonate (4–12 mM) considerably enhanced the photosynthetic efficiency of *Scenedesmus* sp. BHU1 culture in exponential phase, but elevated levels (beyond 12 mM sodium bicarbonate) had little effect. Several studies have been published describing the effects of salinity on photosynthetic activity, which decreased after increasing salinity (Salbitani et al., 2020), which led to the conclusion that salinity induced suppression of Photosystem II (PSII) activity is thought to be produced by impaired PSII repair and lower D1 protein turnover (Allakhverdiev et al., 2002).

The maximum non-photochemical quenching (NPQmax) is caused by excess excitation energy dissipation of the PSII reaction center as heat (Ware et al., 2015). The present NPQmax data show an initial increase in NPQmax with increasing sodium bicarbonate supplementations up to 12 mM followed by a descending order in the range of 16–20 mM sodium bicarbonate. The highest NPQmax was found in culture grown with 12 mM sodium bicarbonate at 10 days (1.399 ± 0.014), which was 2.1-fold higher than the 12 mM sodium bicarbonate at 0 days grown culture (0.643 ± 0.070). Salinity induced PSII reaction center inactivation could explain a decrease in the NPQmax at higher concentrations of sodium bicarbonate (Szabó et al., 2005). Overall, the results suggest that *Scenedesmus* sp. BHU1 with optimum bicarbonate dose results in a significant and quick increase in cell proliferation. These beneficial and dose-dependent positive effects on growth, photosynthetic efficiency, and pigment content suggest that this salt could be a good as inorganic carbon source for lipid accumulation.

### Effect of Sodium Bicarbonate Supplementation on Dry Cell Weight and Biomass Productivity

**Figure 6:**
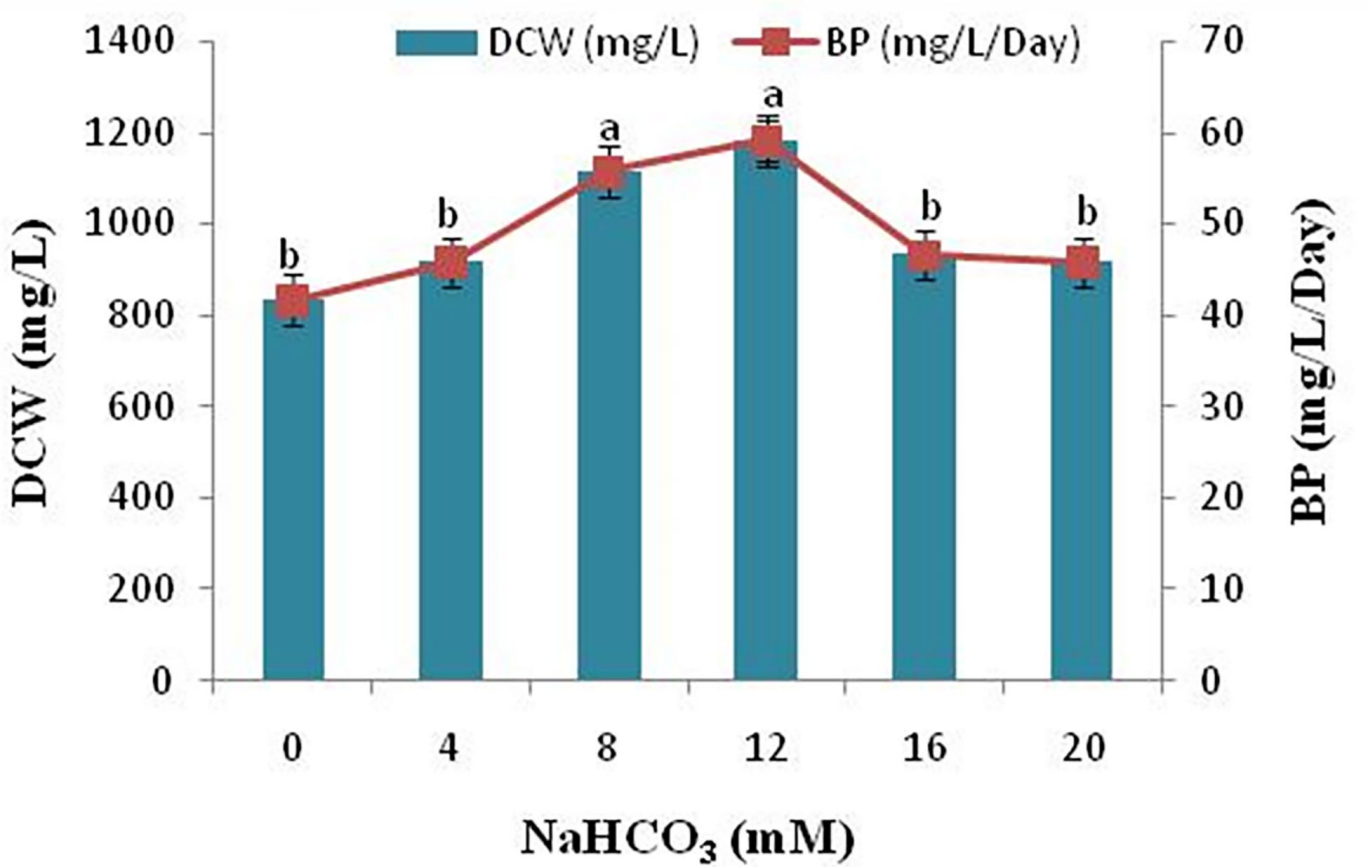
Effect of different sodium bicarbonate concentrations on biomass yield and lipid content of microalga *Scenedesmus* sp. BHU1. Bars are mean ± SE of three replicates. Bars adhered with different alphabets (a and b) are statistically significant at *p* < 0.05 (Tukey’s-b *post hoc* test).

Next to sunlight and water, the most prerequisite resource for photoautotrophic cell growth and lipid synthesis is inorganic carbon. In the present study, addition of sodium bicarbonate to the growth medium significantly increased the dry cell weight (DCW) and biomass productivity (BP) of the microalgae *Scenedesmus* sp. BHU1. The dry cell weight and biomass productivity of the microalga *Scenedesmus* sp. BHU1 has been demonstrated in Figure 6. In general, photoautotrophic microalgae use ambient CO_2_ as a source of inorganic carbon. However, CO_2_ has an exceptionally low solubility rate in water, hence sodium bicarbonate supplementation is used for microalga *Scenedesmus* sp. BHU1 which provide the inorganic carbon required to develop higher biomass. It has been reported that adding sodium bicarbonate as a carbon source to the microalgae growing in culture medium enhances the DCW and biomass of microalgae (White et al., 2013; Pancha et al., 2015). The sodium bicarbonate increased biomass of the *Scenedesmus* sp. BHU1, substantially in this investigation (Figure 6) suggests that supplementing microalgae with sodium bicarbonate improves cell division and metabolism.

Addition of sodium bicarbonate (4–12 mM) enhanced biomass yields dose-dependently, with no considerable difference in DCW and biomass content (*p* < 0.05) detected beyond 12 mM, sodium bicarbonate supplementation because above which it becomes intolerable to the microalgal cells. The DCW and biomass productivity of the microalga *Scenedesmus* sp. BHU1 was significantly reduced in the range of 16–20 mM sodium bicarbonate growing culture. The decline in DCW and biomass of microalgae can be due to the fact that addition of sodium bicarbonate to growing culture leads to a sharp increase in the pH, making the solution alkaline, which is not suitable for growth of microalgae. The highest DCW and biomass content was found with 12 mM sodium bicarbonate (1183.333 ± 44.096 mg/L and 59.167 ± 2.205 mg/L/day) which was 1.4- and 1.4-fold higher than the control grown culture (833.333 ± 33.333 mg/L and 41.667 ± 1.667 mg/L/day). In a similar investigation, the presence of sodium bicarbonate increased biomass yield and lipid content in the green microalga *Scenedesmus* sp. CCNM 1077, *C. vulgaris*, and the diatom *Phaeodactylum tricornutum* (Peng et al., 2014; Pancha et al., 2015; Tu et al., 2018). When *Scenedesmus* sp. CCNM 1077 were treated with 0.6 g/L supplemented sodium bicarbonate the biomass yield was 28.32 mg/L/day (Pancha et al., 2015). In our study, the biomass yield of isolated microalga *Scenedesmus* sp. BHU1 is higher than that of earlier reported isolated strains that have been proposed as greater possible biofuel feedstock. The biomass productivity showed that adding an optimum amount of carbon as sodium bicarbonate can boost the microalgal photosynthetic performance and increasing the rate of reproduction. This study shows that using bicarbonate at the right concentration can boost microalgal development for CO_2_ reduction and biofuel generation.

### Effect of Sodium Bicarbonate Supplementation on Biochemical Composition

**Figure 7:**
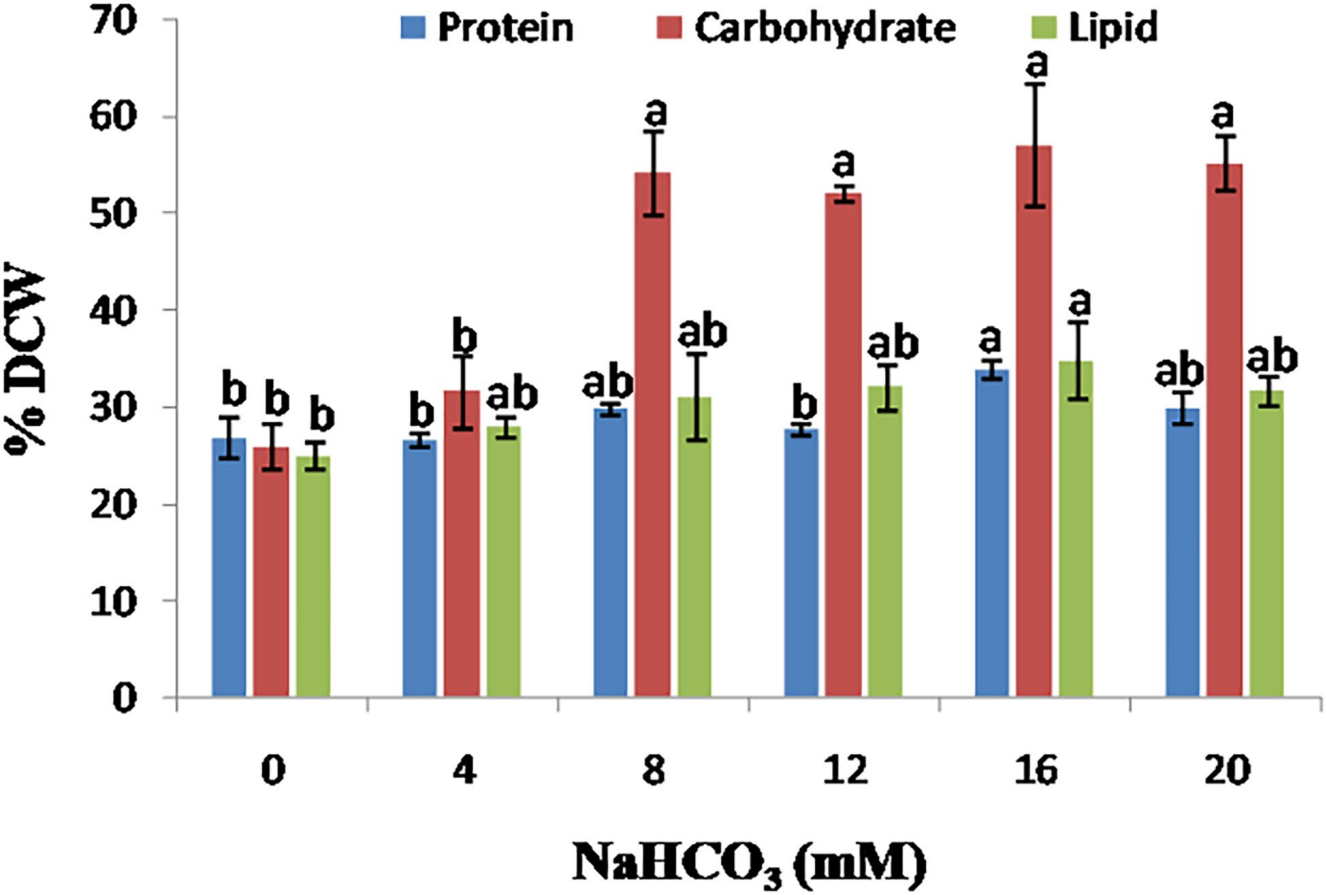
Effect of different sodium bicarbonate concentrations on biochemical composition of microalga *Scenedesmus* sp. BHU1. Bars are mean ± SE of three replicates. Bars adhered with different alphabets (a and b) are statistically significant at *p* < 0.05 (Tukey’s-b *post hoc* test).

In microalgal cells, a reliable supply of inorganic carbon is required for regular photosynthesis, carbon fixation, and the production of biochemical components such as protein, carbohydrate, and lipid. This can be accomplished by supplying CO_2_ gas into the media or adding salts such as sodium bicarbonate. Bicarbonate has a higher solubility than CO_2_ and, hence, has the potential to be employed commercially. The effect of sodium bicarbonate addition on the protein, carbohydrate, and lipid contents of the microalgae *Scenedesmus* sp. BHU1 is shown in Figure 7. Protein content of microalgae *Scenedesmus* sp. BHU1 increased in a dose-dependent manner. In the present study, microalgal cells cultured with 16 mM sodium bicarbonate has the highest protein content (33.782 ± 0.900%), followed by 20 mM (29.752 ± 1.658%). Changing protein content with different sodium bicarbonate supplemented microalgal culture can be advantageous because these proteins can also be utilized to produce biofuels, such as biomethane.

Carbon availability and supply is a critical metabolic regulator in microalgae, governing carbohydrate and lipid synthesis (Fan et al., 2012). The addition of 16 mM sodium bicarbonate to the growth medium increased the carbohydrate content from (25.787 ± 2.366 to 56.920 ± 6.390%), as shown in Figure 7. In the microalgae *Scenedesmus* sp. BHU1, the results show a positive relationship between sodium bicarbonate supplementation and cellular carbohydrate content. It is already reported that high inorganic carbon availability has been linked to increased activity of Ribulose-1, 5-bisphosphate carboxylase/oxygenase (RuBisCO), a crucial enzyme in the conversion of 3-phosphoglycerate, a substrate for carbohydrate and fatty acid production in microalgae (Peng et al., 2014). There was no significant difference (*p* < 0.05) found in carbohydrate content in cells cultivated with 8 mM (54.111 ± 4.355%) and 20 mM (55.047 ± 2.844%) sodium bicarbonate supplemented culture. Because of the high starch content, which can be quickly turned into hexose sugar, several recent studies demonstrate that microalgal carbohydrates are superior to plant-derived carbohydrates. In the present study, the carbohydrate content of leftover de-oiled microalgal biomass was investigated, which was then utilized in biodiesel coproduction, lowering the cost of value-added product generation. In our study, *Scenedesmu*s sp. BHU1 has carbohydrate content (56.920 ± 6.390%) with 16 mM sodium bicarbonate, indicating that this microalga could be a possible source of carbohydrate for biofuel synthesis such as biomethane or other value-added products.

In microalgae *Scenedesmus* sp. BHU1, sodium bicarbonate addition also enhanced the total lipid content along with carbohydrate as shown in Figure 7. There was no significant difference (*p <* 0.05) in total lipid content of microalgal cells cultivated with 8 mM (30.971 ± 4.548%) and 12 mM (31.973 ± 2.336%) sodium bicarbonate supplementation. In the present study, the microalgal cultures treated with 16 mM (34.693 ± 4.001%) sodium bicarbonate have the highest lipid content. When bicarbonate is added to the culture medium, the pH rises, and microalgae modify their membrane composition to cope with the alkaline stress. The biochemical content of green microalgae *Scenedesmus* sp. BHU1 has been compared to previously reported microalgae as shown in Table 2 (Sajjadi et al., 2018). The results suggest that the microalgae *Scenedesmus* sp. BHU1 is a potential strain for biofuel feedstock because of their high carbohydrate and lipid contents.

**Table 2:** The biochemical content of green microalgae *Scenedesmus* sp.

Microalgae strain	Protein (%)	Carbohydrates (%)	Total lipid content (%)
*Ankistrodesmus* sp.	16.24–18.66	4.48–5.97	11.48–31
*Chlamydomonas* sp.	58.8	18.5	22.7
*Chlorella minutissima*	47.89	8.06	14–57
*Chlorella sorokiana*	40.5	26.8	22–24
*Chlorella vulgaris*	51–58	12–17	41–58
*Dunaliella salina*	57	32	6–25
*Dunaliella tertiolecta*	20–29	12.2–14	11–16
*Nannochloropsis oculata*	10–27	17–27	22–29
*Scenedesmus dimorphos*	8–18	21–52	16–40
*Scenedesmus falcatus*	3.37–7.83	2.73–6.83	6.41–9.6
*Scenedesmus protuberans*	25.4–45.05	20.95–29.21	17.53–29.30
*Scenedesmus sp.* CCNM 1077	50.12	25.56	20.91
*Scenedesmus* sp.	29–37	32.7–41	17–24
*Scenedesmus obliquus*	10–45	20–40	30–50
Scenedesmus sp. BHU1	33.782	56.920	34.69

BHU1 has been compared to previously reported microalgae (modified from Sajjadi et al., 2018).

### Lipid Analysis by BODIPY 505/515 and Nile Red Staining at 16 mM Sodium Bicarbonate Supplementation

**Figure 8:**
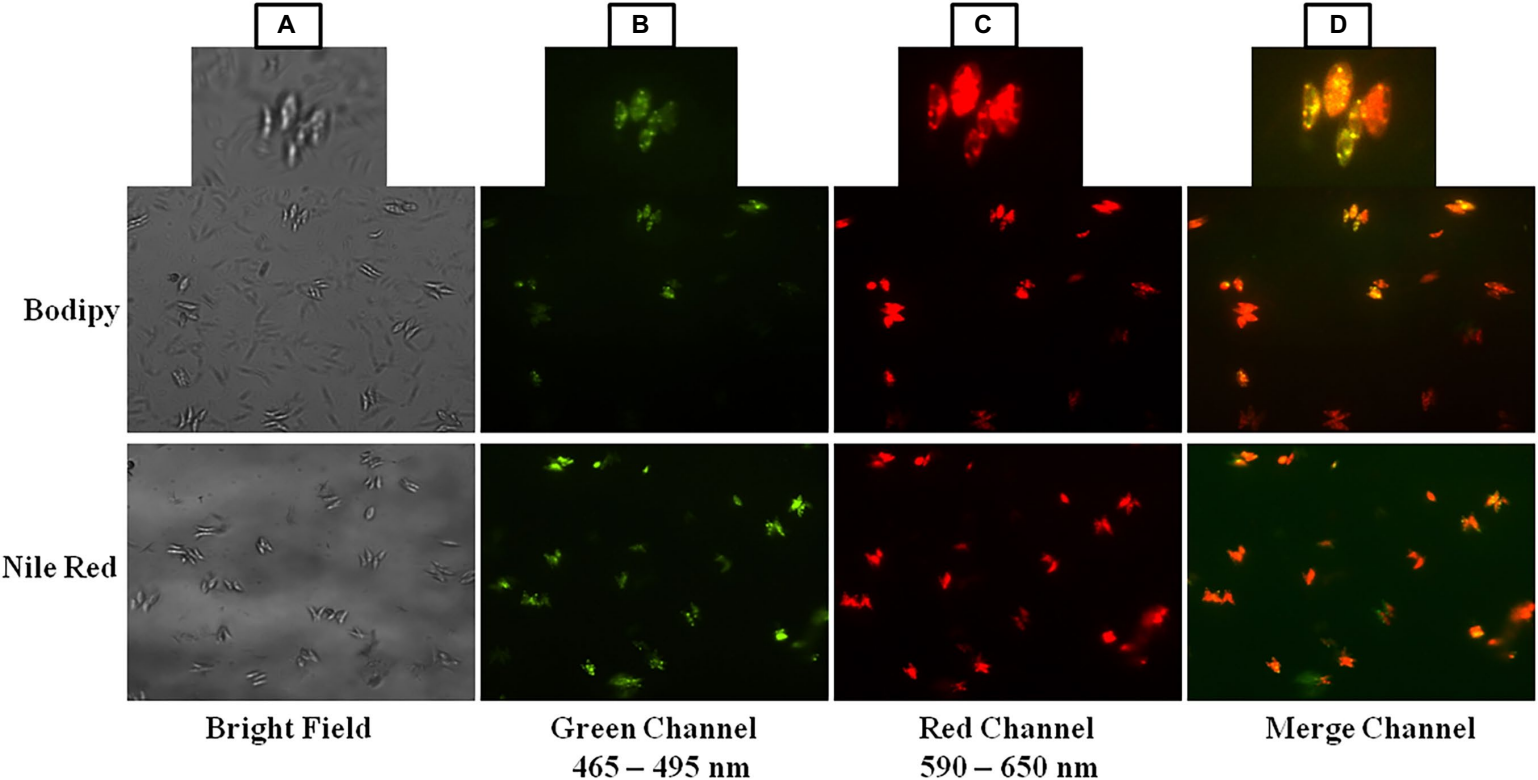
BODIPY 505/515 and Nile Red stained cells of *Scenedesmus* sp. BHU1 under fluorescence microscope in different channels.

BODIPY 505/515 and Nile Red are fluorescent dyes that preferentially stain microalgal cellular lipids (Govender et al., 2012; Sitepu et al., 2012; Katayama et al., 2019). For many years, BODIPY and Nile Red have been used as microalgal lipid probes and their mechanisms are beginning to be understood. The use of BODIPY and Nile Red fluorescence dye to monitor the neutral lipid content of microalgae is a quick method. Under a fluorescence microscope, microalgae labeled with BODIPY and Nile Red were examined for the existence of intracellular lipid droplets. The green, red, and merge channels show dye fluorescence in the presence of cellular lipids of microalga *Scenedesmus* sp. BHU1 (Figures 8B-D) while bright field image does not show any cellular lipid (Figure 8A). The amount of lipid staining dye inside the cell and the size of the neutral lipid droplet determine the degree of the fluorescence. The occurrence of neutral lipids such as triglycerides and hydrocarbons was indicated by a sizable number of algal cells emitting red fluorescence in the field of the red channel between 590 and 650 nm wavelengths (Figure 8C). The presence of a considerable number of significant lipids that synthesizes in the cells of microalga *Scenedesmus* sp. BHU1 was confirmed by the intensity of the BODIPY and Nile Red emitted fluorescence. A remarkably similar study also showed that this fluorescence was found to be significantly linked to neutral lipids in microalgae like *Tetraselmis suecica*, *Nannochloropsis oculate*, and *Scenedesmus* sp. ISTGA1 (Wong et al., 2014; Balduyck et al., 2015; Tripathi et al., 2015). Hence, based on all these observations, the microalga *Scenedesmus* sp. BHU1 is a rich source of lipid which is a good indicator of biofuel feedstock.

## Conclusion

The microalga *Scenedesmus* sp. emerged as a potential carbon uptake aspirant in the form of inorganic carbon, excess carbon is efficiently incorporated into the biomass for use in lipid accumulation and biofuel production. In the current study, the microalga isolated from wastewater habitat of Banaras Hindu University was identified to be *Scenedesmus* sp. BHU1. The growth of the strain BHU1 in terms of an optical density, pigments content, chlorophyll fluorescence, and biomass content was maximum under a surplus inorganic carbon, at 12 mM, sodium bicarbonate supplementation. The microalgal isolate *Scenedesmus* sp. BHU1 accumulated a significant amount of carbohydrate and intracellular neutral lipid as compared to other earlier reported microalgal species. As a result, it is an excellent aspirant for biofuel feedstock. Finally, it can be concluded that microalgal strain from wastewater environment can be beneficial in the field of inorganic carbon uptake and biofuel production. When sodium bicarbonate is added in the growing medium as a carbon source, the pH of the medium rises, inhibiting the growth of microalgae. Furthermore, investigation is required to optimize the pH of a bicarbonate-supplemented medium during Stage I cultivation and nutrient amelioration for Stage II cultivation to stimulate intracellular lipid synthesis, especially when bicarbonate is employed as a partial replacement for gaseous CO_2_.

## Data Availability Statement

The raw data supporting the conclusions of this article will be made available by the authors, without undue reservation.

## Author Contributions

RG and RS designed the study, methodology, and investigation. RG, RS, PY, and AK wrote the manuscript. RG and EA_A acquired funding. RG, RS, and AK supervised the study. A-BA-A, AH, EA_A, and AD revised, edited, and provided valuable feedback to this study. All authors have read and agreed to the published version of the manuscript.

## Funding

The authors would like to extend their sincere appreciation to the Researchers Supporting Project Number (RSP-2021/134), King Saud University, Riyadh, Saudi Arabia.

## Conflict of Interest

The authors declare that the research was conducted in the absence of any commercial or financial relationships that could be construed as a potential conflict of interest.

## Publisher’s Note

All claims expressed in this article are solely those of the authors and do not necessarily represent those of their affiliated organizations, or those of the publisher, the editors and the reviewers. Any product that may be evaluated in this article, or claim that may be made by its manufacturer, is not guaranteed or endorsed by the publisher.

## Abbreviations

Chl-a: Chlorophyll-a
Chl-b: Chlorophyll-b
Fv: Variable chlorophyll fluorescence
Fm: Maximum chlorophyll fluorescence
Fo: Minimum chlorophyll fluorescence
Fv/Fm: Maximum photochemical quantum efficiency
YII: Effective photochemical quantum yield
ETRmax: Maximum electron transport rate
NPQmax: Maximum non-photochemical quenching
PSII: Photosystem II
